# The role of O-GlcNAcylation in development

**DOI:** 10.1242/dev.201370

**Published:** 2023-03-16

**Authors:** Ignacy Czajewski, Daan M. F. van Aalten

**Affiliations:** ^1^ School of Life Sciences, University of Dundee, Dundee DD1 5EH, UK; ^2^ Institute of Molecular Precision Medicine, Xiangya Hospital, Central South University, Changsha 410000, China; ^3^ Department of Molecular Biology and Genetics, University of Aarhus, Aarhus 8000, Denmark

**Keywords:** O-GlcNAcylation, Glycosylation, Polycomb group proteins, Stem cells, Differentiation

## Abstract

O-GlcNAcylation is a dynamic post-translational modification performed by two opposing enzymes: O-GlcNAc transferase and O-GlcNAcase. O-GlcNAcylation is generally believed to act as a metabolic integrator in numerous signalling pathways. The stoichiometry of this modification is tightly controlled throughout all stages of development, with both hypo/hyper O-GlcNAcylation resulting in broad defects. In this Primer, we discuss the role of O-GlcNAcylation in developmental processes from stem cell maintenance and differentiation to cell and tissue morphogenesis.

## Teaser

**Summary:** This Primer introduces the role of O-GlcNAcylation, an essential post-translational modification, in stem cells and development.

## Introduction

**Fig. 1.**
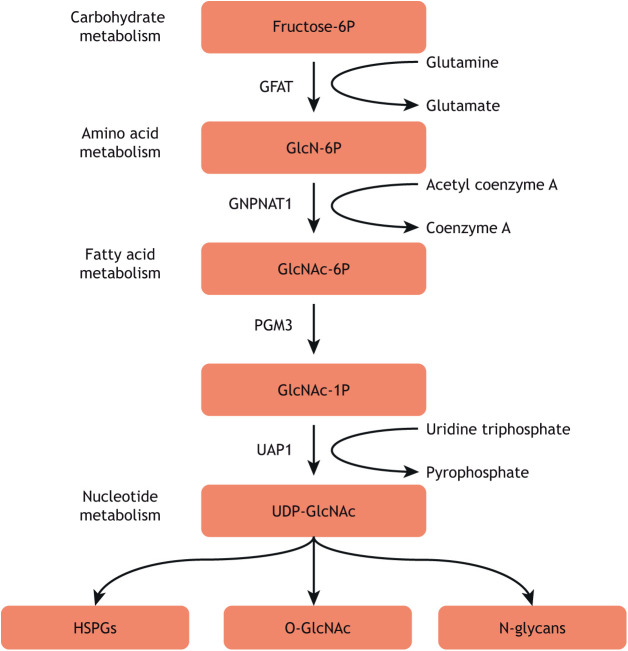
**The hexosamine biosynthetic pathway.** The hexosamine biosynthetic pathway is responsible for the generation of UDP-GlcNAc and is highly conserved across metazoans. The first and rate-limiting enzyme of this pathway is glutamine fructose-6-phosphate amidotransferase (GFAT), followed by glucosamine 6-phosphate N-acetyltransferase (GNPNAT1), phosphoglucomutase 3 (PGM3) and UDP-N-acetylhexosamine pyrophosphorylase (UAP1). This pathway uses 2-3% of glucose (Marshall et al., 1991) in the cell and its product UDP-GlcNAc is a crucial substrate for several types of glycosylation, including O-GlcNAcylation, heparan sulphate proteoglycans (HSPGs) and N-glycans.

Proteins can be post-translationally modified in several different ways, including phosphorylation, ubiquitylation and glycosylation (Khoury et al., 2011; Varki, 2017). O-linked β-N-acetylglucosaminylation (O-GlcNAcylation) is a mono-glycosylation that can occur on serine and threonine residues of nucleocytoplasmic proteins (Torres and Hart, 1984; Holt et al., 1987). Like several other forms of glycosylation, O-GlcNAcylation requires uridine diphosphate N-acetylglucosamine (UDP-GlcNAc) as a substrate for the transfer of the sugar moiety onto proteins (Haltiwanger et al., 1990). UDP-GlcNAc is the product of the highly conserved hexosamine biosynthetic pathway (HBP) and its concentrations are generally believed to depend on the levels of various metabolites in the cell, crucially glucose (Fig. 1) (Marshall et al., 2004; Swamy et al., 2016; Chiaradonna et al., 2018), leading to hypotheses about the role of O-GlcNAcylation as a metabolic integrator. However, this may be a tissue-specific effect because recent research has demonstrated that elevated glucose has no effect on UDP-GlcNAc generation in *ex vivo* heart tissue (Olson et al., 2020). Mechanistically, O-GlcNAc transferase (OGT) is the enzyme that installs the sugar moiety on the target protein (Kreppel et al., 1997; Lubas et al., 1997) and the hydrolase O-GlcNAcase (OGA) catalyses its removal (Heckel et al., 1998; Gao et al., 2001). The presence of both an O-GlcNAc ‘writer’ and ‘eraser’ allows the levels of O-GlcNAcylation to rapidly change throughout development (Liu et al., 2012), as well as in response to changing UDP-GlcNAc concentration (Kreppel and Hart, 1999; Mariappa et al., 2011) and stress conditions, such as elevated temperature (Zachara et al., 2004; Radermacher et al., 2014; Mariappa et al., 2018). Modulation of O-GlcNAcylation is, in part, mediated by post-transcriptional and post-translational regulation of OGT and OGA, although the details of how these mechanisms interplay to control varying O-GlcNAcylation throughout development remain unknown (Whelan et al., 2008; Bullen et al., 2014; Tan et al., 2021). Conversely, an important function of O-GlcNAcylation is the regulation of other post-translational modifications (PTMs), such as ubiquitylation (Ruan et al., 2013; Chen et al., 2021) and phosphorylation (Hart et al., 2011; Bauer et al., 2015), occurring either on or near otherwise modified residues (Kamemura et al., 2002; White et al., 2020), or regulating PTM writers (Dias et al., 2009) and erasers (Liu et al., 2020).

This Primer summarises the current state of knowledge on the role of O-GlcNAcylation in development from loss-of-function studies in various species, highlighting roles in gene regulation and cell signalling. We also discuss emerging themes in this field, including the roles of O-GlcNAcylation in nervous system development and in integrating metabolism with signalling pathways and transcriptional regulation.

## O-GlcNAc and loss-of-function studies

### O-GlcNAc transferase

In most metazoans, a lack of functional OGT protein is developmentally lethal. This was first observed in *Drosophila*, in which zygotic homozygosity for amorphic alleles of the gene encoding OGT, *super sex combs* (*sxc*), results in lethality at the pupal stage of development (Ingham, 1984; Gambetta et al., 2009; Sinclair et al., 2009). However, in the absence of maternally contributed *sxc* gene product, lethality in *sxc*-null *Drosophila* occurs late in embryonic development or during early larval stages (Ingham, 1984; Gambetta and Müller, 2014). In vertebrates, the importance of OGT is more pronounced. Even in culture, mammalian cells cannot survive in the absence of *Ogt* and the loss of OGT protein is embryonically lethal in mice (Shafi et al., 2000; O'Donnell et al., 2004). Due to this lethality upon global loss of OGT, several groups have attempted to characterise the function of OGT in individual cell types using tissue-specific knockouts. While many conditional knockouts of *Ogt* result in lethality (Cheng et al., 2020; Xiong et al., 2022), this approach has demonstrated the far-ranging roles of the gene in development (Table 1). For example, the excision of *Ogt* in neuronal stem cells results in broad cortical malformations (Cheng et al., 2020), while conditional knockout of the gene in kidney podocytes results in defects in postnatal maturation of these cells (Ono et al., 2017).

**Table 1.**
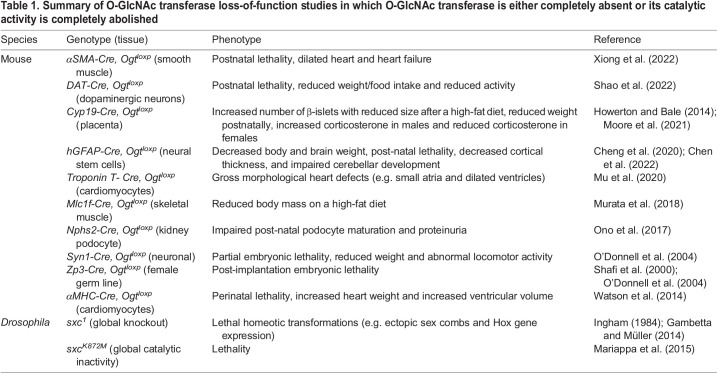
Summary of O-GlcNAc transferase loss-of-function studies in which O-GlcNAc transferase is either completely absent or its catalytic activity is completely abolished

It has been recently shown that several individuals with intellectual disability (ID) carry mutations in *OGT*, with family pedigree analysis convincingly indicating the causality of the mutations (Niranjan et al., 2015; Vaidyanathan et al., 2017; Willems et al., 2017; Pravata et al., 2019; Pravata, et al., 2020a). The disorder, termed OGT-linked congenital disorder of glycosylation (OGT-CDG), presents with a range of clinical signs beyond ID that are generally indicative of broad developmental defects, including clinodactyly (curved fingers), short stature and facial dysmorphisms (Pravata et al., 2020b). Several causal *OGT* mutations in ID do not result in measurable decreases in global O-GlcNAcylation in patient-derived fibroblasts (Willems et al., 2017) or when modelled in mouse embryonic stem cells (mESCs) (Pravata et al., 2019), due to compensatory mechanisms, such as OGA downregulation.

### O-GlcNAcase

In contrast to OGT deficiency, the lack of OGA appears to be better tolerated by most animals (Table 2). Flies lacking OGA are fully viable and fertile, presenting with only a mild phenotypes, such as larger adult size, a semi-penetrant oogenesis defect (Akan et al., 2016, 2021), mild neuronal and behavioural phenotypes (Muha et al., 2020), and increased intestinal stem cell proliferation (Na et al., 2020). Unlike in *Drosophila*, the lack of OGA or its catalytic activity in mice results in broad developmental defects in several organs and pre/perinatal lethality (Table 2) (Yang et al., 2012; Muha et al., 2021). Mice lacking OGA present with reduced body weight as early as embryonic day (E)14.5 (Yang et al., 2012), along with reduced lung alveolar space (Yang et al., 2012), reduced brain size along with a relative increase in ventricle size (Muha et al., 2021), as well as defects in glycogen storage and mobilisation, which are hypothesised to be key contributors to perinatal lethality in the absence of OGA function (Keembiyehetty et al., 2015).

**Table 2.**
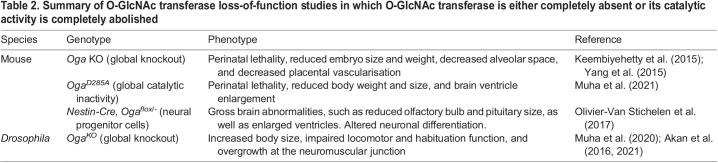
Summary of O-GlcNAc transferase loss-of-function studies in which O-GlcNAc transferase is either completely absent or its catalytic activity is completely abolished

These findings underscore the importance of maintaining appropriate O-GlcNAcylation throughout development, as even slight perturbations to O-GlcNAc homeostasis, as seen in OGT-CDG, can result in pronounced effects. With over 5000 proteins in the human proteome identified as O-GlcNAc modified, some of these modified multiple sites (Wulff-Fuentes et al., 2021); it remains a major challenge to identify functionally important sites and their role in developmental processes.

## O-GlcNAcylation in embryonic stem cells

**Fig. 2.**
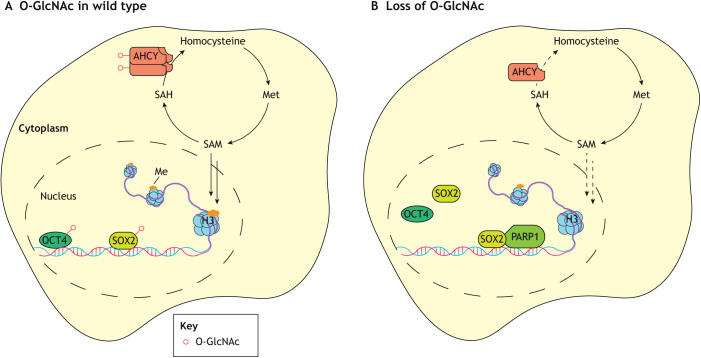
**Role of O-GlcNAcylation in embryonic stem cell maintenance.** (A) Mouse embryonic stem cells (mESCs) with normal O-GlcNAcylation (O-GlcNAc) are maintained by a variety of transcriptional regulators. Some of the most important, the Yamanaka factors, can be used to induce pluripotency in terminally differentiated cells. Among these are OCT4 and SOX2, both of which are functionally O-GlcNAc modified. OCT4 transcriptional activity is regulated by O-GlcNAc in both its DNA-binding and transactivation domains, with increased O-GlcNAc promoting transcriptional activity. The effects of O-GlcNAc of SOX2 are more site specific: although both occur in the transactivation domain, SOX2 O-GlcNAcylation can either increase its transcriptional activity or change its DNA occupancy by altering its interactions with other proteins important in maintenance of stemness. Histone (H3) methylation (Me) is also important for perpetuating stemness. A crucial metabolic pathway for maintaining appropriate levels of methylation is the methionine (Met) cycle that produces S-adenosyl methionine (SAM), a donor substrate for methyltransferases. O-GlcNAc plays a crucial role in this cycle by enhancing the activity of adenosylhomocysteinase (AHCY), which converts S-adenosylhomocysteine to homocysteine and adenosine. (B) Loss of O-GlcNAcylation disrupts transcriptional regulation, e.g. by changing SOX2 protein interactions with co-factors such as poly [ADP-ribose] polymerase 1 (PARP1), which results in differentiation.

As mentioned above, O-GlcNAcylation is essential from the earliest stages of development and OGT is essential even in mESCs (Shafi et al., 2000). However, beyond being required for general cell viability, O-GlcNAcylation plays an important role in maintaining proliferation and pluripotency (Fig. 2). In mESCs, elevating O-GlcNAcylation by inhibiting OGA delays differentiation, while decreasing O-GlcNAcylation through knockdown of OGT impairs stem cell self-renewal (Jang et al., 2012; Speakman et al., 2014). The effects of O-GlcNAcylation on mESC maintenance appear to occur through the modulation of several pathways, including core pluripotency factors (such as Oct4 and Sox2), auxiliary factors [such as estrogen-related receptor β (ESRRB), phosphokinase PKCζ and proteasome activator subunit 3 (Psme3)] and epigenetic regulators (Jang et al., 2012; Shi et al., 2013; Miura et al., 2018; Hao et al., 2019; Pecori et al., 2021).

### Pluripotency factor targets of O-GlcNAcylation

The effects of O-GlcNAcylation on protein function have largely been revealed by amino acid substitutions of serine/threonine residues that act as O-GlcNAc target sites. For example, alanine mutagenesis of O-GlcNAc sites in the DNA-binding domain of both mouse and human Oct4 (T228A and T235A, respectively) reduces Oct4 transcriptional activity in mouse embryonic fibroblasts and human cancer cells, respectively (Jang et al., 2012; Constable et al., 2017). Evidence that this effect is due to O-GlcNAcylation (and not to other post-translational modifications of Oct4 that occur in the same region) comes from the observation that OGT overexpression increases Oct4 transcriptional activity (Jang et al., 2012; Constable et al., 2017). Similarly, O-GlcNAcylation of serine 25 of ESRRB promotes pluripotency and self-renewal through increasing the stability and transcriptional activity of the protein (Hao et al., 2019). O-GlcNAcylation also directly modulates protein-protein interactions between Oct4 and ESSRB, as well as other regulators of stemness (Hao et al., 2019). However, in human cells, the effects of O-GlcNAcylation on Oct4 and ESRRB function have primarily been observed in cancer cells. Although additional research in human embryonic stem cells is required to understand the role of O-GlcNAcylation on these two proteins, the effects of O-GlcNAcylation on Oct4 transcriptional activity are not conserved in human embryonic stem cells, casting doubt on the importance of this mechanism in human development (Constable et al., 2017).

Whereas O-GlcNAcylation of Oct4 and ESRRB appears to promote stemness (Jang et al., 2012; Constable et al., 2017; Hao et al., 2019), Sox2 O-GlcNAcylation has mixed effects on maintaining pluripotency and self-renewal, depending on the site modified (Myers et al., 2016; Kim et al., 2021). Sox2 O-GlcNAcylation occurs in the transactivation domain and two neighbouring sites have been the focus of recent studies (Myers et al., 2016; Kim et al., 2021). Alanine mutagenesis of Sox2 threonine 258 increases the expression of early differentiation genes and reduces ectodermal marker genes in mESCs, indicating that threonine 258 O-GlcNAcylation is required for the function of Sox2 in self-renewal and regulation of the expression of ectoderm-lineage genes (Kim et al., 2021). Conversely, O-GlcNAcylation of Sox2 at serine 248 decreases its self-renewal activity and alanine mutagenesis of this site increases the reprogramming efficiency of somatic cells to induced pluripotent stem cells (Myers et al., 2016). O-GlcNAcylation of Sox2 at serine 248 impairs its interaction with poly ADP-ribose polymerase 1 (Parp1), a protein known to cooperatively regulate stemness, and thus Sox2 genomic binding (Lai et al., 2012). Given the antagonistic effects of O-GlcNAcylation of these two adjacent sites, the regulation of their modification and relative importance during development remains a pertinent question.

### O-GlcNAcylation and epigenetics in pluripotency

In addition to directly controlling transcription factors involved in pluripotency, epigenetic markers required for stem cell maintenance are regulated by O-GlcNAcylation. In particular, DNA and histone methylation are sensitive to OGT activity because methylation modification enzymes and enzymes in the metabolic pathway that generates the most common methyl donor for methylation, S-adenosylmethionine (SAM), are regulated by O-GlcNAcylation (Fig. 2). Depletion of adenosylhomocysteinase (AHCY), a key enzyme in the SAM-generating methionine cycle, reduces the expression of pluripotency markers and increases the expression of differentiation markers through the loss of histone 3 lysine 4 (H3K4) methylation (Zhu et al., 2020). AHCY is O-GlcNAcylated on threonine 136, increasing its catalytic efficiency by almost an order of magnitude through facilitating AHCY homotetramerization and thereby promoting stem cell maintenance (Zhu et al., 2020). O-GlcNAcylation also regulates the function of histone methyltransferases directly (Chu et al., 2014; Lo et al., 2018; Parween et al., 2022), although this function has been investigated more extensively in the context of differentiation and so is discussed in greater detail later.

In the context of the role of DNA methylation in stem cell maintenance, the interplay between the TET (Ten-eleven translocation) family of proteins and OGT has been a focus of research. TET proteins (TET1-TET3) are key enzymes responsible for demethylating DNA through converting 5-methylcytosine (5mC) to 5-hydroxymethylcytosine and in so doing regulate gene expression (Tahiliani et al., 2009). TET1 and TET2 are particularly enriched in mESCs, and a loss of protein function results in earlier differentiation (Ito et al., 2010; Shi et al., 2013). The loss of self-renewal and increased differentiation upon knockdown of TET1 and/or TET2 is likely due to their role in derepression (by hydroxylating 5mC) of core pluripotency factors, primarily Nanog (Ito et al., 2010). One of the first functions ascribed to OGT/TET interactions was the stabilising effect of O-GlcNAcylation on TET1 (Shi et al., 2013); however, more recent research shows a more-complex relationship between OGT and the TET family of proteins. TET1 activity is regulated by its interaction with OGT, with OGT binding-deficient TET1 unable to rescue defects in hematopoietic stem cell production in TET knockout zebrafish (Hrit et al., 2018). The mechanistic details of how OGT and O-GlcNAcylation affect stability and activity of TET proteins remain to be fully understood, although extensive crosstalk between O-GlcNAcylation and phosphorylation occurs on TET proteins (Bauer et al., 2015). TET proteins have also been found to regulate OGT activity, promoting O-GlcNAcylation of chromatin-associated protein complexes and histones (Chen et al., 2013; Deplus et al., 2013). For example, both TET2 and TET3 promote O-GlcNAcylation of host cell factor 1 (HCF1), which regulates the integrity of the H3K4 methyltransferase complex SET1/COMPASS, as well as its binding to chromatin (Deplus et al., 2013). Although the consequences of this interaction on stem cell maintenance have not been investigated, SET1 is known to promote mESC colony formation, potentially through its interaction with Oct4, enhancing its activity (Fang et al., 2016).

It is important to note that, despite the high degree of conservation of O-GlcNAc sites on the pluripotency factors mentioned here, the phenotypic effects of perturbations in O-GlcNAc addition and removal on pluripotency maintenance in human pluripotent stem cells are milder relative to mouse cells (Maury et al., 2013; Andres et al., 2017). Human embryonic and induced pluripotent stem cells are unperturbed by OGA or OGT manipulation, although increasing or decreasing O-GlcNAcylation does accelerate their rate of differentiation (Maury et al., 2013; Andres et al., 2017). Whether this is a result of broader cellular differences between mouse and human pluripotent stem cells (Schnerch et al., 2010) or of differences in the role of O-GlcNAcylation on target proteins remains to be fully explored.

## O-GlcNAcylation in differentiation and cell fate determination

The first function ascribed to OGT (before its molecular characterisation) was its role as a polycomb group (PcG) protein (Ingham, 1984). More recently, the diverse roles of O-GlcNAcylation in differentiation and cell fate determination have become more extensively investigated. The importance of O-GlcNAcylation in neuronal development is particularly pronounced; however, many unanswered questions remain regarding mechanisms regulated by this PTM, across different germ layers.

### OGT as a polycomb group gene

**Fig. 3.**
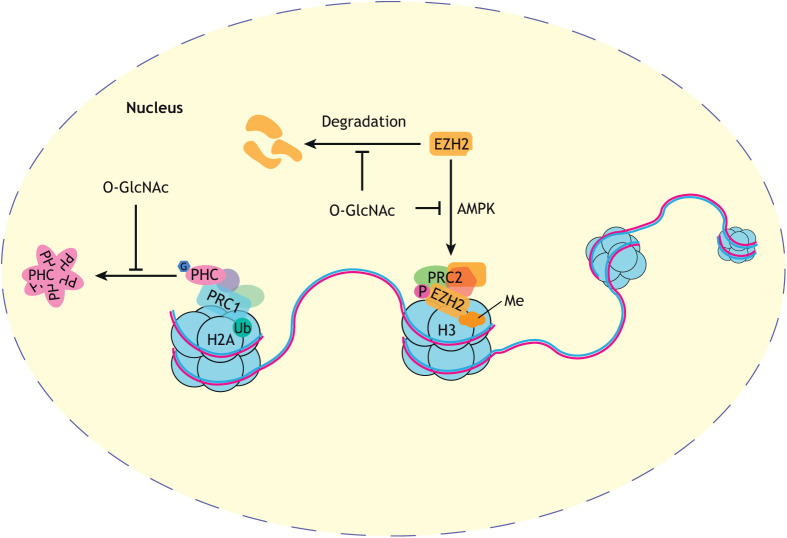
**O-GlcNAcylation in PcG protein function.** O-GlcNAcylation (O-GlcNAc) is important for the functioning of several PcG proteins; notably, preventing the aggregation of polyhomeotic (PHC), a component of polycomb repressive complex 1 (PRC1), which catalyses the ubiquitylation (Ub) of histone 2A (H2A). In histone 3 (H3) methylating (Me) polycomb repressive complex 2 (PRC2), O-GlcNAc appears to play an important role in preventing the incorporation of enhancer of zeste homolog 2 (EZH2) into the complex by preventing its phosphorylation by AMP-activated protein kinase (AMPK). However, in addition to this role, when not in complex with other components of PRC2, O-GlcNAc prevents the degradation of EZH2. G, O-GlcNAc; P, phosphorylation.

Proteins encoded by PcG genes are important transcriptional regulators that prevent ectopic gene expression during development. Notable targets of PcG silencing are the homeotic Hox genes, making PcG proteins central to the maintenance of cell identity in the anterior-posterior axis (Struhl and Akam, 1985; Golbabapour et al., 2013). PcG proteins assemble into histone-modifying complexes that act in concert to maintain gene repression at loci adjacent to genomic regions called polycomb response elements (PREs) (Muller and Bienz, 1991; Horard et al., 2000; Kassis and Brown, 2013). In *Drosophila*, O-GlcNAcylation prevents the aggregation of a stoichiometric component of polycomb repressive complex 1 (PRC1), Polyhomeotic (Ph) (Fig. 3) (Gambetta and Müller, 2014). In the absence of OGT activity, or the serine/threonine-rich domain of Ph that is O-GlcNAc modified, Ph forms aggregates that prevent PRC1-mediated maintenance of Hox gene repression, such as *ultrabithorax* (Ubx) (Gambetta and Müller, 2014). Although it has not been investigated whether this observation translates to vertebrates, human orthologs of Ph (PHC2 and PHC3) also aggregate *in vitro* in the absence of O-GlcNAcylation (Gambetta and Müller, 2014). In human embryonic stem cells, the catalytic subunit of PRC1 (RING1B) is also modified by O-GlcNAc, altering its chromosomal distribution (Maury et al., 2015): O-GlcNAcylated RING1B is enriched at genes implicated in neuronal differentiation and development, relative to the unmodified protein (Maury et al., 2015). O-GlcNAcylation decreases upon neuronal differentiation of human ESCs (Maury et al., 2013); therefore, the loss of RING1B O-GlcNAcylation may be important for the derepression of genes involved in neuronal differentiation.

In addition to PRC1*,* early genetic interaction studies proposed a role for OGT, and by extension O-GlcNAcylation, in the function of other PcG complexes. In *Drosophila, sxc* amorphic alleles enhance homeotic transformations as a result of mutations in elements of either the polycomb repressive complex 2 (PRC2) or the polycomb repressive-deubiquitylase complex (PR-DUB) (Campbell et al., 1995). Recent research has demonstrated the molecular functions of O-GlcNAcylation in these complexes in mammalian systems. The primary focus has been on the histone methyltransferase subunit enhancer of zeste homologue 2 (EZH2) of PRC2 (Fig. 3). *In vitro*, O-GlcNAcylation increases EZH2 stability, specifically when this protein is not in complex with other PRC2 components, and enhances its histone methyltransferase activity (Chu et al., 2014; Lo et al., 2018). However, *in vivo* research indicates that elevated O-GlcNAcylation can also negatively regulate EZH2 function. In a rat model of maternal hyperglycaemia, a condition leading to elevated embryonic O-GlcNAcylation, EZH2 has increased phosphorylation at threonine 311 (Parween et al., 2022). Phosphorylation at this site hinders the association between EZH2 and SUZ12, another PRC2 component (Wan et al., 2018). The consequent deregulation of PRC2 activity results in misexpression of neurogenic genes, causing elevated neuronal marker expression at earlier stages of development, a phenotype that can be reversed by OGT inhibition (Parween et al., 2022). However, how O-GlcNAcylation positively regulates phosphorylation of EZH2 remains unknown. Threonine 311 of EZH2 is phosphorylated by AMP-activated protein kinase (AMPK) (Wan et al., 2018), which is likely more active when O-GlcNAcylated in specific contexts (Bullen et al., 2014; Xu et al., 2014). However, it remains unknown whether this mechanism is responsible for the phenotypes seen in hyperglycaemic mouse embryos, or whether other mechanisms of O-GlcNAc/phosphorylation crosstalk are involved. The effects of O-GlcNAcylation on EZH2 may also, in part, explain why human iPSCs acquire neuronal markers earlier in differentiation in the presence of OGA inhibitors (Parween et al., 2017).

The component of the PR-DUB that genetically interacts with *sxc* is Additional sex combs (*Asx*) (Milne et al., 1999). *Asx* is important for the functioning of both PcG and the opposing trithorax group (TrxG) proteins, and, therefore, plays a role in both restricting and promoting the expression of Hox genes (Milne et al., 1999; Li et al., 2017). All the human orthologs of *Asx* are O-GlcNAcylated (Wulff-Fuentes et al., 2021), with O-GlcNAcylation stabilising the ortholog Asx-like 1 (ASXL1) (Inoue et al., 2018). The functional significance of the relationship between OGT and ASXL1 remains understudied; however, some evidence indicates that reduced O-GlcNAcylation of ASXL1 results in deficient myeloid differentiation by inhibiting H3K4 methylation (Inoue et al., 2018).

### O-GlcNAc in cell fate determination

Several studies have found that the precise regulation of O-GlcNAcylation is required for cells to differentiate into a specific fate. Several cell types, such as cardiomyocytes (Kim et al., 2009), myocytes (Ogawa et al., 2012), keratinocytes (Sohn et al., 2014), neutrophils and erythroid cells (Zhang et al., 2019), require reduced levels of O-GlcNAcylation to acquire their cell fate, while several others, such as osteoblasts (Koyama and Kamemura, 2015), adipocytes (Ishihara et al., 2010) and chondrocytes (Andrés-Bergós et al., 2012), require increased levels of O-GlcNAcylation to differentiate. In some cases, the inability of cells to acquire a specific identity upon O-GlcNAc deregulation may simply reflect the role of O-GlcNAcylation in maintaining stemness. However, important roles for O-GlcNAcylation in the function of cell fate-determining transcription factors have also been identified, such as Sp1 (Yang et al., 2001; Sohn et al., 2014). In keratinocytes, upon differentiation, global levels of O-GlcNAcylation decrease, including O-GlcNAcylation of Sp1. This results in a de-repression of Sp1 transcriptional activity, which increases the expression of genes encoding structural proteins important for the formation of the protective outer layer of the epidermis: the stratum corneum (Sohn et al., 2014).

### O-GlcNAc and neural development

The role of O-GlcNAcylation in neuronal differentiation has been of particular interest in recent years due to its relevance to OGT-CDG. An increase in mESC O-GlcNAcylation impairs differentiation towards a neuronal cell fate and mice lacking *Oga* in neuronal precursor cells present with increased numbers of immature neurons (Olivier-Van Stichelen et al., 2017). Conversely, the loss of O-GlcNAcylation in neural stem cells (NSCs) through genetic ablation of *OGT* using Cre-Lox recombination or knockdown with short hairpin RNA (shRNA) results in a depletion of these cells and impairs neurogenesis (Cheng et al., 2020; White et al., 2020; Chen et al., 2021; Shen et al., 2021), although reports of mechanisms causing this phenotype are conflicting. One explanation for impaired neurogenesis in the absence of OGT is via STAT3, a key transcription factor that promotes a glial cell fate (Cao et al., 2010; Hong and Song, 2014). O-GlcNAcylation of STAT3 prevents its phosphorylation on a nearby residue, thereby inhibiting its transcriptional activity (White et al., 2020). Although this effect has primarily been investigated in the context of ageing, it also extends to NSCs isolated from postnatal day (P) 1 mice. More recent research challenges the notion that O-GlcNAcylation impairs differentiation towards a neural fate and suggests that impaired neurogenesis occurs primarily because of NSC depletion. Specifically, shRNA knockdown of OGT in mouse embryos results in the preferential acquisition of a neuronal cell fate by transfected cells, an effect that can also be observed in human forebrain organoids (Shen et al., 2021). This shift towards a neuronal cell fate is accompanied by a reduction in the number of proliferating NSCs, explaining the reduced total number of differentiated neurons upon loss of OGT. Further supporting this mechanism of impaired neurogenesis, loss of OGT in mouse adult NSCs reduces proliferation and results in increased differentiation towards a neuronal cell fate, at the expense of glial differentiation (Chen et al., 2021). Mechanistically, this shift towards a neuronal cell fate can be explained by reduced levels of either β-catenin or reduced Notch signalling (Chen et al., 2021; Shen et al., 2021). Disentangling how such mechanisms may interact in the developing brain is crucial for the understanding conditions such as OGT-CDG or hyperglycaemia-induced neural tube defects (Kim et al., 2017).

## O-GlcNAcylation in cell and tissue morphogenesis

Beyond controlling stem cell proliferation, differentiation and cell identity, OGT activity can also modulate morphogenesis. For example, in mouse embryos from hyperglycaemic dams, defects in neural tube closure can be reversed by OGT inhibition (Kim et al., 2017). In zebrafish, *ogt* overexpression disrupts epiboly and microtubule formation, resulting in thicker shorter microtubules (Webster et al., 2009). However, the mechanistic roles of O-GlcNAcylation in these phenotypes has not been further investigated.

### Signalling pathways

More-detailed insights into the role of O-GlcNAcylation in morphogenesis have been gained from research in *Drosophila*, from genetic disruption of components of the HBP. For example, loss of *nesthocker* (*nst*), the *Drosophila* ortholog of phosphoglucomutase 3 (*PGM3*), results in defective cell migration of mesodermal cells during gastrulation and later defective tracheal formation (Mariappa et al., 2011). These defects are a result of altered fibroblast growth factor (FGF) signalling, wherein a crucial adaptor protein, Downstream of FGFR (Dof), requires O-GlcNAcylation for appropriate function. This is illustrated by phenotypic rescue of *nst* mutants by overexpression of a chimeric receptor retaining the FGF receptor extracellular domain while the intracellular domain is substituted for a tyrosine kinase that is not dependent on Dof for signal transduction. Although mechanistic details of the role of O-GlcNAcylation on Dof are not understood, the lack of additional research in this area is likely due to Dof not being conserved in mammals (Mariappa et al., 2011).

In addition to FGF signalling, O-GlcNAc is also linked to bone morphogenetic protein (BMP) signalling. In *Drosophila*, excessive activity of the BMP ligand decapentaplegic (Dpp) manifests most visibly as a tissue morphogenesis phenotype, with dorsal puckering from excess dorsal movement of epithelial cells. This phenotype is also observed in *sxc* mutant embryos and when the HBP is disrupted, e.g. in *Drosophila* that are homozygous for a hypomorphic allele of the gene *Mummy* (*mmy*) (Moulton et al., 2020). *mmy* is the ortholog of the human gene UDP-N-acetylglucosamine pyrophosphorylase (*UAP1*) (Humphreys et al., 2013), which encodes an enzyme that catalyses the formation of UDP-GlcNAc from N-acetylglucosamine-1-phosphate and UTP – the final step in the HBP (Mio et al., 1998) (Fig. 1). Although proteomics approaches have identified that some elements of the Dpp transcriptional activator complex (AP-1) are O-GlcNAc modified (Selvan et al., 2017), the phenotypes seen in *mmy* mutants are more likely conveyed through defective O-GlcNAcylation of the type I BMP receptor saxophone (Sax) (Moulton et al., 2020). In the absence of functional OGT, the *Drosophila* SMAD ortholog, Mothers against dpp (Mad), is ectopically phosphorylated by Sax, causing the dorsalising phenotypes seen in *mmy* and *sxc* mutant embryos. Interestingly, embryos with wild-type *sxc* from mothers fed a sugar-free diet present with a similar phenotype to embryos homozygous for an amorphic allele of *sxc*, demonstrating that the role of O-GlcNAcylation in conveying metabolic signals in development is conserved from *Drosophila* to mammals (Moulton et al., 2020). Whether this particular mechanism is conserved in vertebrates remains to be investigated, although the putatively modified region of Sax is conserved in its human orthologs (Moulton et al., 2020).

### Neurite morphology

Another understudied function of O-GlcNAcylation is its role in neurite outgrowth and axonal branching. OGA overexpression in chicken embryonic forebrain neurons is associated with increased primary axon length and the number of neurons entering a branching program (Francisco et al., 2009), whereas knockout of OGT in mouse embryonic cortical neurons reduces their total axonal length (Cheng et al., 2020). The mechanism by which loss of O-GlcNAcylation promotes axon branching is hypothesised to be downstream of protein kinase A (PKA) because PKA activation increases axon branching and this is prevented when OGA is inhibited (Francisco et al., 2009); however, specific conveyors have not been further investigated. O-GlcNAcylation also affects dendrite outgrowth in cultured hippocampal and cortical neurons (Cheng et al., 2020; Shen et al., 2021), with neurite outgrowth of the former rescued by overexpression of β-catenin (Shen et al., 2021), which is reduced in cells lacking OGT. β-Catenin is stabilised by O-GlcNAcylation (Olivier-Van Stichelen et al., 2014); therefore, it is likely that the effects of reduced O-GlcNAcylation on neurite outgrowth are directly mediated by this role of OGT. Similar defects have been observed in models of OGT-CDG. In *Drosophila*, both loss of O-GlcNAcase and mutations in *sxc* that are analogous to those seen in individuals with ID result in disrupted neuromuscular junction formation (Muha et al., 2020; Fenckova et al., 2022), while neurons differentiated from mESCs carrying a patient mutation are characterised by reduced neurite outgrowth (Pravata et al., 2019).

## Concluding remarks

In summary, O-GlcNAcylation exerts broad effects on development by modulating a variety of cellular processes, including transcriptional regulation, epigenetics and metabolic pathways. The role of O-GlcNAcylation in these processes is diverse, although key among them is the crosstalk of O-GlcNAcylation with other PTMs. An emerging theme in the role of O-GlcNAc in development is its connection to metabolic states, in part mediated through the HBP (Mariappa et al., 2011; Humphreys et al., 2013; Moulton et al., 2020; Wong et al., 2020). In humans, defective maternal metabolic homeostasis, such as poorly managed pregestational diabetes mellitus, can also affect development (Ornoy et al., 2015). Interestingly, much like mutations in *OGT*, some of the most pronounced clinical manifestations of maternal pregestational diabetes mellitus during development affect the central nervous system, including neural tube defects and autism (Ornoy et al., 2015). Initial studies have shown that maternal hyperglycaemia can negatively impact embryonic development, both through altering embryonic (Kim et al., 2017) and placental O-GlcNAcylation (Dela Justina et al., 2017, 2018). The study of O-GlcNAcylation in development may lead to crucial discoveries related to the effects of metabolic states on development, particularly relevant in the light of an increase in individuals affected by metabolic disorders (Saklayen, 2018).

## References

[DEV201370C1] Akan, I., Love, D. C., Harwood, K. R., Bond, M. R. and Hanover, J. A. (2016). Drosophila O-GlcNAcase deletion globally perturbs chromatin O-GlcNAcylation. *J. Biol. Chem.* 291, 9906-9919. 10.1074/jbc.M115.704783 26957542 PMC4858994

[DEV201370C2] Akan, I., Halim, A., Vakhrushev, S. Y., Clausen, H. and Hanover, J. A. (2021). Drosophila O-GlcNAcase mutants reveal an expanded glycoproteome and novel growth and longevity phenotypes. *Cells* 10, 1026. 10.3390/cells10051026 33925313 PMC8145559

[DEV201370C3] Andrés-Bergós, J., Tardio, L., Larranaga-Vera, A., Gómez, R., Herrero-Beaumont, G. and Largo, R. (2012). The increase in O-linked N-acetylglucosamine protein modification stimulates chondrogenic differentiation both in vitro and in vivo. *J. Biol. Chem.* 287, 33615-33628. 10.1074/jbc.M112.354241 22859309 PMC3460460

[DEV201370C4] Andres, L. M., Blong, I. W., Evans, A. C., Rumachik, N. G., Yamaguchi, T., Pham, N. D., Thompson, P., Kohler, J. J. and Bertozzi, C. R. (2017). Chemical modulation of protein O-GlcNAcylation via OGT inhibition promotes human neural cell differentiation. *ACS Chem. Biol.* 12, 2030-2039. 10.1021/acschembio.7b00232 28541657 PMC5850955

[DEV201370C5] Bauer, C., Göbel, K., Nagaraj, N., Colantuoni, C., Wang, M., Müller, U., Kremmer, E., Rottach, A. and Leonhardt, H. (2015). Phosphorylation of TET proteins is regulated via O-GlcNAcylation by the O-Linked N-Acetylglucosamine transferase (OGT). *J. Biol. Chem.* 290, 4801-4812. 10.1074/jbc.M114.605881 25568311 PMC4335217

[DEV201370C6] Bullen, J. W., Balsbaugh, J. L., Chanda, D., Shabanowitz, J., Hunt, D. F., Neumann, D. and Hart, G. W. (2014). Cross-talk between two essential nutrient-sensitive enzymes O-GlcNAc Transferase (OGT) and Amp-Activated Protein Kinase (AMPK). *J. Biol. Chem.* 289, 10592-10606. 10.1074/jbc.M113.523068 24563466 PMC4036179

[DEV201370C7] Campbell, R. B., Sinclair, D. A. R., Couling, M. and Brock, H. W. (1995). Genetic interactions and dosage effects of Polycomb group genes of Drosophila. *Mol. Gen. Genet.* 246, 291-300. 10.1007/BF00288601 7854314

[DEV201370C8] Cao, F., Hata, R., Zhu, P., Nakashiro, K. I. and Sakanaka, M. (2010). Conditional deletion of Stat3 promotes neurogenesis and inhibits astrogliogenesis in neural stem cells. *Biochem. Biophys. Res. Commun.* 394, 843-847. 10.1016/j.bbrc.2010.03.092 20303333

[DEV201370C9] Chen, Q., Chen, Y., Bian, C., Fujiki, R. and Yu, X. (2013). TET2 promotes histone O-GlcNAcylation during gene transcription. *Nature* 493, 561-564. 10.1038/nature11742 23222540 PMC3684361

[DEV201370C10] Chen, J., Dong, X., Cheng, X., Zhu, Q., Zhang, J., Li, Q., Huang, X., Wang, M., Li, L., Guo, W. et al. (2021). Ogt controls neural stem/progenitor cell pool and adult neurogenesis through modulating Notch signaling. *Cell Rep.* 34, 108905. 10.1016/j.celrep.2021.108905 33789105

[DEV201370C11] Chen, L., Li, Y., Song, Z., Xue, S., Liu, F., Chang, X., Wu, Y., Duan, X. and Wu, H. (2022). O-GlcNAcylation promotes cerebellum development and medulloblastoma oncogenesis via SHH signaling. *Proc. Natl. Acad. Sci. USA* 119, e2202821119. 10.1073/pnas.2202821119 35969743 PMC9407465

[DEV201370C12] Cheng, J., Wu, Y., Chen, L., Li, Y., Liu, F., Shao, J., Huang, M., Fan, M. and Wu, H. (2020). Loss of O-GlcNAc transferase in neural stem cells impairs corticogenesis. *Biochem. Biophys. Res. Commun.* 532, 541-547. 10.1016/j.bbrc.2020.08.084 32896380

[DEV201370C13] Chiaradonna, F., Ricciardiello, F. and Palorini, R. (2018). The nutrient-sensing hexosamine biosynthetic pathway as the hub of cancer metabolic rewiring. *Cells* 7, 53. 10.3390/cells7060053 29865240 PMC6025041

[DEV201370C14] Chu, C. S., Lo, P. W., Yeh, Y. H., Hsu, P. H., Peng, S. H., Teng, Y. C., Kang, M. L., Wong, C. H. and Juan, L. J. (2014). O-GlcNAcylation regulates EZH2 protein stability and function. *Proc. Natil. Acad. Sci. USA* 111, 1355-1360. 10.1073/pnas.1323226111 PMC3910655 24474760

[DEV201370C15] Constable, S., Lim, J. M., Vaidyanathan, K. and Wells, L. (2017). O-GlcNAc transferase regulates transcriptional activity of human Oct4. *Glycobiology* 27, 927-937. 10.1093/glycob/cwx055 28922739 PMC6410957

[DEV201370C16] Dela Justina, V., Gonçalves, J. S., De Freitas, R. A., Fonseca, A. D., Volpato, G. T., Tostes, R. C., Carneiro, F. S., Lima, V. V. and Giachini, F. R. (2017). Increased O-linked N-acetylglucosamine modification of NF-ΚB and augmented cytokine production in the placentas from hyperglycemic rats. *Inflammation* 40, 1773-1781. 10.1007/s10753-017-0620-7 28688099

[DEV201370C17] Dela Justina, V., Dos Passos Junior, R. R., Bressan, A. F., Tostes, R. C., Carneiro, F. S., Soares, T. S., Volpato, G. T., Lima, V. V., Martin, S. S. and Giachini, F. R. (2018). O-linked N-acetyl-glucosamine deposition in placental proteins varies according to maternal glycemic levels. *Life Sci.* 205, 18-25. 10.1016/j.lfs.2018.05.013 29746846

[DEV201370C18] Deplus, R., Delatte, B., Schwinn, M. K., Defrance, M., Méndez, J., Murphy, N., Dawson, M. A., Volkmar, M., Putmans, P., Calonne, E., et al. (2013). TET2 and TET3 regulate GlcNAcylation and H3K4 methylation through OGT and SET1/COMPASS. *EMBO J.* 32, 645-655. 10.1038/emboj.2012.357 23353889 PMC3590984

[DEV201370C19] Dias, W. B., Cheung, W. D., Wang, Z. and Hart, G. W. (2009). Regulation of calcium/calmodulin-dependent kinase IV by *O*-GlcNAc modification. *J. Biol. Chem.* 284, 21327-21337. 10.1074/jbc.M109.007310 19506079 PMC2755857

[DEV201370C20] Fang, L., Zhang, J., Zhang, H., Yang, X., Jin, X., Zhang, L., Skalnik, D. G., Jin, Y., Zhang, Y., Huang, X., et al. (2016). H3K4 methyltransferase Set1a is a key Oct4 coactivator essential for generation of Oct4 positive inner cell mass. *Stem Cells* 34, 565-580. 10.1002/stem.2250 26785054

[DEV201370C21] Fenckova, M., Muha, V., Mariappa, D., Catinozzi, M., Czajewski, I., Blok, L. E. R., Ferenbach, A. T., Storkebaum, E., Schenck, A. and Van Aalten, D. M. F. (2022). Intellectual disability-associated disruption of O-GlcNAc cycling impairs habituation learning in Drosophila. *PLOS Genet.* 18, e1010159. 10.1371/journal.pgen.1010159 35500025 PMC9140282

[DEV201370C22] Francisco, H., Kollins, K., Varghis, N., Vocadlo, D., Vosseller, K. and Gallo, G. (2009). O-GLcNAc post-translational modifications regulate the entry of neurons into an axon branching program. *Dev. Neurobiol.* 69, 162-173. 10.1002/dneu.20695 19086029 PMC2747243

[DEV201370C23] Gambetta, M. C. and Müller, J. (2014). O-GlcNAcylation prevents aggregation of the polycomb group repressor polyhomeotic. *Dev. Cell* 31, 629-639. 10.1016/j.devcel.2014.10.020 25468754

[DEV201370C24] Gambetta, M. C., Oktaba, K. and Müller, J. (2009). Essential role of the glycosyltransferase Sxc/Ogt in polycomb repression. *Science* 325, 93-96. 10.1126/science.1169727 19478141

[DEV201370C25] Gao, Y., Wells, L., Comer, F. I., Parker, G. J. and Hart, G. W. (2001). Dynamic O-glycosylation of nuclear and cytosolic proteins: cloning and characterization of a neutral, cytosolic beta-N-acetylglucosaminidase from human brain. *J. Biol. Chem* 276, 9838-9845. 10.1074/jbc.M010420200 11148210

[DEV201370C201] Golbabapour, S., Majid, N. A., Hassandarvish, P., Hajrezaie, M., Abdulla, M. A. and Hadi, A. H. A. (2013). Gene silencing and polycomb group proteins: An overview of their structure, mechanisms and phylogenetics. *OMICS* 17, 283-296. 10.1089/omi.2012.0105 23692361 PMC3662373

[DEV201370C26] Haltiwanger, R. S., Holt, G. D. and Hart, G. W. (1990). Enzymatic addition of O-GlcNAc to nuclear and cytoplasmic proteins. Identification of a uridine diphospho-N-acetylglucosamine:peptide beta-N-acetylglucosaminyltransferase. *J. Biol. Chem.* 265, 2563-2568. 10.1016/S0021-9258(19)39838-2 2137449

[DEV201370C27] Hao, Y., Fan, X., Shi, Y., Zhang, C., Sun, D.e, Qin, K., Qin, W., Zhou, W. and Chen, X. (2019). Next-generation unnatural monosaccharides reveal that ESRRB O-GlcNAcylation regulates pluripotency of mouse embryonic stem cells. *Nat. Commun.* 10, 4065. 10.1038/s41467-018-07882-8 31492838 PMC6731260

[DEV201370C28] Hart, G. W., Slawson, C., Ramirez-Correa, G. and Lagerlof, O. (2011). Cross Talk between O-GlcNAcylation and phosphorylation: roles in signaling, transcription, and chronic disease. *Annu. Rev. Biochem.* 80, 825-858. 10.1146/annurev-biochem-060608-102511 21391816 PMC3294376

[DEV201370C29] Heckel, D., Comtesse, N., Brass, N., Blin, N., Zang, K. D. and Meese, E. (1998). Novel immunogenic antigen homologous to hyaluronidase in meningioma. *Hum. Mol. Genet.* 7, 1859-1872. 10.1093/hmg/7.12.1859 9811929

[DEV201370C30] Holt, G. D., Snow, C. M., Senior, A., Haltiwanger, R. S., Gerace, L. and Hart, G. W. (1987). Nuclear pore complex glycoproteins contain cytoplasmically disposed O-linked N-acetylglucosamine. *J. Cell Biol.* 104, 1157-1164. 10.1083/jcb.104.5.1157 3571327 PMC2114481

[DEV201370C31] Hong, S. and Song, M. R. (2014). STAT3 but not STAT1 is required for astrocyte differentiation. *PLoS One* 9, e86851. 10.1371/journal.pone.0086851 24466267 PMC3900679

[DEV201370C202] Horard, B., Tatout, C., Poux, S. and Pirrotta, V. (2000). Structure of a Polycomb Response Element and in vitro binding of polycomb group complexes containing GAGA factor. *Mol. Cell. Biol.* 20, 3187-3197. 10.1128/mcb.20.9.3187-3197.2000 10757803 PMC85613

[DEV201370C32] Howerton, C. L. and Bale, T. L. (2014). Targeted placental deletion of OGT recapitulates the prenatal stress phenotype including hypothalamic mitochondrial dysfunction. *Proc. Natl. Acad. Sci. USA* 111, 9639-9644. 10.1073/pnas.1401203111 24979775 PMC4084439

[DEV201370C33] Hrit, J., Goodrich, L., Li, C., Wang, B. A., Nie, J., Cui, X., Martin, E. A., Simental, E., Fernandez, J., Liu, M. Y., et al. (2018). OGT binds a conserved C-terminal domain of TET1 to regulate TET1 activity and function in development. *eLife* 7, e34870. 10.7554/eLife.34870 30325306 PMC6214653

[DEV201370C34] Humphreys, G. B., Jud, M. C., Monroe, K. M., Kimball, S. S., Higley, M., Shipley, D., Vrablik, M. C., Bates, K. L. and Letsou, A. (2013). Mummy, A UDP-N-acetylglucosamine pyrophosphorylase, modulates DPP signaling in the embryonic epidermis of Drosophila. *Dev. Biol.* 381, 434-445. 10.1016/j.ydbio.2013.06.006 23796903 PMC3775589

[DEV201370C35] Ingham, P. W. (1984). A gene that regulates the bithorax complex differentially in larval and adult cells of Drosophila. *Cell* 37, 815-823. 10.1016/0092-8674(84)90416-1 6430566

[DEV201370C36] Inoue, D., Fujino, T., Sheridan, P., Zhang, Y. Z., Nagase, R., Horikawa, S., Li, Z., Matsui, H., Kanai, A., Saika, M., et al. (2018). A novel ASXL1-OGT axis plays roles in H3K4 methylation and tumor suppression in myeloid malignancies. *Leukemia* 32, 1327-1337. 10.1038/s41375-018-0083-3 29556021

[DEV201370C37] Ishihara, K., Takahashi, I., Tsuchiya, Y., Hasegawa, M. and Kamemura, K. (2010). Characteristic increase in nucleocytoplasmic protein glycosylation by O-GlcNAc in 3T3-L1 adipocyte differentiation. *Biochem. Biophys. Res. Commun.* 398, 489-494. 10.1016/j.bbrc.2010.06.105 20599697

[DEV201370C38] Ito, S., D'Aalessio, A. C., Taranova, O. V., Hong, K., Sowers, L. C. and Zhang, Y. (2010). Role of Tet proteins in 5mC to 5hmC conversion, ES-cell self-renewal and inner cell mass specification. *Nature* 466, 1129-1133. 10.1038/nature09303 20639862 PMC3491567

[DEV201370C39] Jang, H., Kim, T. W., Yoon, S., Choi, S.-Y., Kang, T.-W., Kim, S.-Y., Kwon, Y.-W., Cho, E.-J. and Youn, H.-D. (2012). O-GlcNAc regulates pluripotency and reprogramming by directly acting on core components of the pluripotency network. *Cell Stem Cell* 11, 62-74. 10.1016/j.stem.2012.03.001 22608532

[DEV201370C40] Kamemura, K., Hayes, B. K., Comer, F. I. and Hart, G. W. (2002). Dynamic interplay between O-glycosylation and O-phosphorylation of nucleocytoplasmic proteins: alternative glycosylation/phosphorylation of Thr-58, a known mutational hot spot of c-Myc in lymphomas, is regulated by mitogens. *J. Biol. Chem.* 277, 19229-19235. 10.1074/jbc.M201729200 11904304

[DEV201370C200] Kassis, J. A. and Brown, J. L. (2013). Polycomb Group Response Elements in Drosophila and vertebrates. *Adv. Genet.* 81, 83-118. 10.1016/B978-0-12-407677-8.00003-8 23419717 PMC4157523

[DEV201370C41] Keembiyehetty, C., Love, D. C., Harwood, K. R., Gavrilova, O., Comly, M. E. and Hanover, J. A. (2015). Conditional knock-out reveals a requirement for O-Linked N-Acetylglucosaminase (O-GlcNAcase) in metabolic homeostasis. *J. Biol. Chem.* 290, 7097-7113. 10.1074/jbc.M114.617779 25596529 PMC4358131

[DEV201370C42] Khoury, G. A., Baliban, R. C. and Floudas, C. A. (2011). Proteome-wide post-translational modification statistics: frequency analysis and curation of the swiss-prot database. *Sci. Rep.* 1, 90. 10.1038/srep00090 22034591 PMC3201773

[DEV201370C43] Kim, H. S., Park, S. Y., Choi, Y. R., Kang, J. G., Joo, H. J., Moon, W. K. and Cho, J. W. (2009). Excessive O-GlcNAcylation of proteins suppresses spontaneous cardiogenesis in ES cells. *FEBS Lett.* 583, 2474-2478. 10.1016/j.febslet.2009.06.052 19591829

[DEV201370C44] Kim, G., Cao, L., Reece, E. A. and Zhao, Z. (2017). Impact of protein O-GlcNAcylation on neural tube malformation in diabetic embryopathy. *Sci. Rep.* 7, 11107. 10.1038/s41598-017-11655-6 28894244 PMC5593976

[DEV201370C45] Kim, D. K., Lee, J. S., Lee, E. Y., Jang, H., Han, S., Kim, H. Y., Hwang, I. Y., Choi, J. W., Shin, H. M., You, H. J., et al. (2021). O-GlcNAcylation of Sox2 at threonine 258 regulates the self-renewal and early cell fate of embryonic stem cells. *Exp. Mol. Med.* 53, 1759-1768. 10.1038/s12276-021-00707-7 34819616 PMC8639819

[DEV201370C46] Koyama, T. and Kamemura, K. (2015). Global increase in O-linked N-acetylglucosamine modification promotes osteoblast differentiation. *Exp. Cell Res.* 338, 194-202. 10.1016/j.yexcr.2015.08.009 26302267

[DEV201370C47] Kreppel, L. K. and Hart, G. W. (1999). Regulation of a cytosolic and nuclear O-GlcNAc transferase. Role of the tetratricopeptide repeats. *J. Biol. Chem.* 274, 32015-32022. 10.1074/jbc.274.45.32015 10542233

[DEV201370C48] Kreppel, L. K., Blomberg, M. A. and Hart, G. W. (1997). Dynamic glycosylation of nuclear and cytosolic proteins: cloning and characterization of a unique O-GlcNAc transferase with multiple tetratricopeptide repeats. *J. Biol. Chem.* 272, 9308-9315. 10.1074/jbc.272.14.9308 9083067

[DEV201370C49] Lai, Y. S., Chang, C. W., Pawlik, K. M., Zhou, D., Renfrow, M. B. and Townes, T. M. (2012). SRY (sex determining region Y)-box2 (Sox2)/poly ADP-ribose polymerase 1 (Parp1) complexes regulate pluripotency. *Proc. Natl. Acad. Sci. USA* 109, 3772-3777. 10.1073/pnas.1108595109 22362888 PMC3309723

[DEV201370C50] Li, T., Hodgson, J. W., Petruk, S., Mazo, A. and Brock, H. W. (2017). Additional sex combs interacts with enhancer of zeste and trithorax and modulates levels of trimethylation on histone H3K4 and H3K27 during transcription of hsp70. *Epigenetics & Chromatin* 10, 43. 10.1186/s13072-017-0151-3 28927461 PMC5605996

[DEV201370C51] Liu, Y., Li, X., Yu, Y., Shi, J., Liang, Z., Run, X., Li, Y., Dai, C., Grundke-Iqbal, I., Iqbal, K., et al. (2012). Developmental regulation of protein O-GlcNAcylation, O-GlcNAc transferase, and O-GlcNAcase in mammalian brain. *PLoS One* 7, e43724. 10.1371/journal.pone.0043724 22928023 PMC3425547

[DEV201370C52] Liu, C., Shi, Y., Li, J., Liu, X., Xiahou, Z., Tan, Z., Chen, X. and Li, J. (2020). O-GlcNAcylation of myosin phosphatase targeting subunit 1 (MYPT1) dictates timely disjunction of centrosomes. *J. Biol. Chem.* 295, 7341-7349. 10.1074/jbc.RA119.012401 32295844 PMC7247298

[DEV201370C53] Lo, P. W., Shie, J. J., Chen, C. H., Wu, C. Y., Hsu, T. L. and Wong, C. H. (2018). O-GlcNAcylation regulates the stability and enzymatic activity of the histone methyltransferase EZH2. *Proc. Natl. Acad. Sci. USA* 115, 7302-7307. 10.1073/pnas.1801850115 29941599 PMC6048490

[DEV201370C54] Lubas, W. A., Frank, D. W., Krause, M. and Hanover, J. A. (1997). O-linked GlcNAc transferase is a conserved nucleocytoplasmic protein containing tetratricopeptide repeats. *J. Biol. Chem.* 272, 9316-9324. 10.1074/jbc.272.14.9316 9083068

[DEV201370C55] Mariappa, D., Sauert, K., Mariño, K., Turnock, D., Webster, R., Van Aalten, D. M. F. F., Ferguson, M. A. J. J., Müller, H. A. J., Marino, K., Turnock, D., et al. (2011). Protein O-GlcNAcylation is required for fibroblast growth factor signaling in Drosophila. *Sci. Signal.* 4, ra89. 10.1126/scisignal.2002335 22375049 PMC3660836

[DEV201370C56] Mariappa, D., Zheng, X., Schimpl, M., Raimi, O., Ferenbach, A. T., Müller, H. A. J. and Van Aalten, D. M. F. (2015). Dual functionality of O-GlcNAc transferase is required for Drosophila development. *Open Biol.* 5, 150234. 10.1098/rsob.150234 26674417 PMC4703063

[DEV201370C57] Mariappa, D., Ferenbach, A. T. and Van Aalten, D. M. F. (2018). Effects of hypo- *O*-GlcNAcylation on *Drosophila* development. *J. Biol. Chem.* 293, 7209-7221. 10.1074/jbc.RA118.002580 29588363 PMC5950000

[DEV201370C58] Marshall, S., Bacote, V. and Traxinger, R. R. (1991). Discovery of a metabolic pathway mediating glucose-induced desensitization of the glucose transport system: role of hexosamine in the induction of insulin resistance. *J. Biol. Chem.* 266, 4706-4712. 10.1016/S0021-9258(19)67706-9 2002019

[DEV201370C59] Marshall, S., Nadeau, O. and Yamasaki, K. (2004). Dynamic actions of glucose and glucosamine on hexosamine biosynthesis in isolated adipocytes: differential effects on glucosamine 6-phosphate, UDP-N-acetylglucosamine, and ATP levels. *J. Biol. Chem.* 279, 35313-35319. 10.1074/jbc.M404133200 15199059

[DEV201370C60] Maury, J. J. P., Chan, K. K. K., Zheng, L., Bardor, M. and Choo, A. B. H. (2013). Excess of O-linked N-acetylglucosamine modifies human pluripotent stem cell differentiation. *Stem Cell Res.* 11, 926-937. 10.1016/j.scr.2013.06.004 23859804

[DEV201370C61] Maury, J. J. P., El Farran, C. A., Ng, D., Loh, Y. H., Bi, X., Bardor, M. and Choo, A. B. H. (2015). RING1B O-GlcNAcylation regulates gene targeting of polycomb repressive complex 1 in human embryonic stem cells. *Stem Cell Res.* 15, 182-189. 10.1016/j.scr.2015.06.007 26100231

[DEV201370C62] Milne, T. A., Sinclair, D. A. R. and Brock, H. W. (1999). The Additional sex combs gene of Drosophila is required for activation and repression of homeotic loci, and interacts specifically with Polycomb and super sex combs. *Mol. Gen. Genet.* 261, 753-761. 10.1007/s004380050018 10394912

[DEV201370C63] Mio, T., Yabe, T., Arisawa, M. and Yamada-Okabe, H. (1998). The eukaryotic UDP-N-acetylglucosamine pyrophosphorylases: gene cloning, protein expression, and catalytic mechanism. *J. Biol. Chem.* 273, 14392-14397. 10.1074/jbc.273.23.14392 9603950

[DEV201370C64] Miura, T., Kume, M., Kawamura, T., Yamamoto, K., Hamakubo, T. and Nishihara, S. (2018). O-GlcNAc on PKCζ Inhibits the FGF4-PKCζ-MEK-ERK1/2 pathway via inhibition of PKCζ phosphorylation in mouse embryonic stem cells. *Stem Cell Rep.* 10, 272-286. 10.1016/j.stemcr.2017.11.007 PMC5768893 29249667

[DEV201370C65] Moore, M., Avula, N., Jo, S., Beetch, M. and Alejandro, E. U. (2021). Disruption of O-linked N-acetylglucosamine signaling in placenta induces insulin sensitivity in female offspring. *Int. J. Mol. Sci.* 22, 6918. 10.3390/ijms22136918 34203166 PMC8267851

[DEV201370C66] Moulton, M. J., Humphreys, G. B., Kim, A. and Letsou, A. (2020). O-GlcNAcylation dampens Dpp/BMP signaling to ensure proper drosophila embryonic development. *Dev. Cell* 53, 330-343.e3. 10.1016/j.devcel.2020.04.001 32369743

[DEV201370C67] Mu, Y., Yu, H., Wu, T., Zhang, J., Evans, S. M. and Chen, J. (2020). O-linked β-N-acetylglucosamine transferase plays an essential role in heart development through regulating angiopoietin-1. *PLoS Genet.* 16, e1008730. 10.1371/journal.pgen.1008730 32251422 PMC7182263

[DEV201370C68] Muha, V., Fenckova, M., Ferenbach, A. T., Catinozzi, M., Eidhof, I., Storkebaum, E., Schenck, A. and Van Aalten, D. M. F. (2020). O-GlcNAcase contributes to cognitive function in Drosophila. *J. Biol. Chem.* 295, 8636-8646. 10.1074/jbc.RA119.010312 32094227 PMC7324509

[DEV201370C69] Muha, V., Authier, F., Szoke-Kovacs, Z., Johnson, S., Gallagher, J., Mcneilly, A., Mccrimmon, R. J., Teboul, L. and Van Aalten, D. M. F. (2021). Loss of O-GlcNAcase catalytic activity leads to defects in mouse embryogenesis. *J. Biol. Chem.* 296, 100439. 10.1016/j.jbc.2021.100439 33610549 PMC7988489

[DEV201370C203] Muller, J. and Bienz, M. (1991). Long range repression conferring boundaries of Ultrabithorax expression in the Drosophila embryo. *EMBO J.* 10, 3147-3155. 10.1002/j.1460-2075.1991.tb04876.x 1680676 PMC453036

[DEV201370C70] Murata, K., Morino, K., Ida, S., Ohashi, N., Lemecha, M., Park, S. Y., Ishikado, A., Kume, S., Choi, C. S., Sekine, O., et al. (2018). Lack of O-GlcNAcylation enhances exercise-dependent glucose utilization potentially through AMP-activated protein kinase activation in skeletal muscle. *Biochem. Biophys. Res. Commun.* 495, 2098-2104. 10.1016/j.bbrc.2017.12.081 29253568

[DEV201370C71] Myers, S. A., Peddada, S., Chatterjee, N., Friedrich, T., Tomoda, K., Krings, G., Thomas, S., Maynard, J., Broeker, M., Thomson, M., et al. (2016). SOX2 O-GlcNAcylation alters its protein-protein interactions and genomic occupancy to modulate gene expression in pluripotent cells. *eLife* 5, e10647. 10.7554/eLife.10647 26949256 PMC4841768

[DEV201370C72] Na, H. J.j, Akan, I., Abramowitz, L. K. and Hanover, J. A. (2020). Nutrient-driven O-GlcNAcylation controls DNA damage repair signaling and stem/progenitor cell homeostasis. *Cell Rep.* 31, 107632. 10.1016/j.celrep.2020.107632 32402277 PMC9340802

[DEV201370C73] Niranjan, T. S., Skinner, C., May, M., Turner, T., Rose, R., Stevenson, R., Schwartz, C. E. and Wang, T. (2015). Affected kindred analysis of human X chromosome exomes to identify novel X-linked intellectual disability genes. *PLoS One* 10, e0116454. 10.1371/journal.pone.0116454 25679214 PMC4332666

[DEV201370C74] O'Donnell, N., Zachara, N. E., Hart, G. W. and Marth, J. D. (2004). Ogt-dependent X-chromosome-linked protein glycosylation is a requisite modification in somatic cell function and embryo viability. *Mol. Cell. Biol.* 24, 1680-1690. 10.1128/MCB.24.4.1680-1690.2004 14749383 PMC344186

[DEV201370C75] Ogawa, M., Mizofuchi, H., Kobayashi, Y., Tsuzuki, G., Yamamoto, M., Wada, S. and Kamemura, K. (2012). Terminal differentiation program of skeletal myogenesis is negatively regulated by O-GlcNAc glycosylation. *Biochim. Biophys. Acta Gen. Subj.* 1820, 24-32. 10.1016/j.bbagen.2011.10.011 22056510

[DEV201370C76] Olivier-Van Stichelen, S., Dehennaut, V., Buzy, A., Zachayus, J.-L., Guinez, C., Mir, A.-M., El Yazidi-Belkoura, I., Copin, M.-C., Boureme, D., Loyaux, D., et al. (2014). O-GlcNAcylation stabilizes β-catenin through direct competition with phosphorylation at threonine 41. *FASEB J.* 28, 3325-3338. 10.1096/fj.13-243535 24744147 PMC4101651

[DEV201370C77] Olivier-Van Stichelen, S., Wang, P., Comly, M., Love, D. C. and Hanover, J. A. (2017). Nutrient-driven O-linked N-acetylglucosamine (O-GlcNAc) cycling impacts neurodevelopmental timing and metabolism. *J. Biol. Chem.* 292, 6076-6085. 10.1074/jbc.M116.774042 28246173 PMC5391740

[DEV201370C78] Olson, A. K., Bouchard, B., Zhu, W. Z., Chatham, J. C. and Des Rosiers, C. (2020). First characterization of glucose flux through the hexosamine biosynthesis pathway (HBP) in ex vivo mouse heart. *J. Biol. Chem.* 295, 2018-2033. 10.1074/jbc.RA119.010565 31915250 PMC7029105

[DEV201370C79] Ono, S., Kume, S., Yasuda-Yamahara, M., Yamahara, K., Takeda, N., Chin-Kanasaki, M., Araki, H., Sekine, O., Yokoi, H., Mukoyama, M., et al. (2017). O-linked β-N-acetylglucosamine modification of proteins is essential for foot process maturation and survival in podocytes. *Nephrol. Dial. Transplant.* 32, 1477-1487. 10.1093/ndt/gfw463 28339907

[DEV201370C80] Ornoy, A., Reece, E. A., Pavlinkova, G., Kappen, C. and Miller, R. K. (2015). Effect of maternal diabetes on the embryo, fetus, and children: congenital anomalies, genetic and epigenetic changes and developmental outcomes. *Birth Defects Res.* 105, 53-72. 10.1002/bdrc.21090 25783684

[DEV201370C82] Parween, S., Varghese, D. S., Ardah, M. T., Prabakaran, A. D., Mensah-Brown, E., Emerald, B. S. and Ansari, S. A. (2017). Higher O-GlcNAc levels are associated with defects in progenitor proliferation and premature neuronal differentiation during *in-vitro* human embryonic cortical neurogenesis. *Front. Cell. Neurosci.* 11, 415. 10.3389/fncel.2017.00415 29311838 PMC5742625

[DEV201370C83] Parween, S., Alawathugoda, T. T., Prabakaran, A. D., Dheen, S. T., Morse, R. H., Emerald, B. S. and Ansari, S. A. (2022). Nutrient sensitive protein *O*-GlcNAcylation modulates the transcriptome through epigenetic mechanisms during embryonic neurogenesis. *Life Sci. Alliance* 5, e202201385. 10.26508/lsa.202201385 35470239 PMC9039347

[DEV201370C84] Pecori, F., Kondo, N., Ogura, C., Miura, T., Kume, M., Minamijima, Y., Yamamoto, K. and Nishihara, S. (2021). Site-specific O-GlcNAcylation of Psme3 maintains mouse stem cell pluripotency by impairing P-body homeostasis. *Cell Rep.* 36, 109361. 10.1016/j.celrep.2021.109361 34260942

[DEV201370C85] Pravata, V. M., Muha, V., Gundogdu, M., Ferenbach, A. T., Kakade, P. S., Vandadi, V., Wilmes, A. C., Borodkin, V. S., Joss, S., Stavridis, M. P., et al. (2019). Catalytic deficiency of O-GlcNAc transferase leads to X-linked intellectual disability. *Proc. Natl. Acad. Sci. USA* 116, 14961-14970. 10.1073/pnas.1900065116 31296563 PMC6660750

[DEV201370C86] Pravata, V. M., Gundogdu, M., Bartual, S. G., Ferenbach, A. T., Stavridis, M., Õunap, K., Pajusalu, S., Žordania, R., Wojcik, M. H. and Van Aalten, D. M. F. (2020a). A missense mutation in the catalytic domain of O-GlcNAc transferase links perturbations in protein O-GlcNAcylation to X-linked intellectual disability. *FEBS Lett.* 594, 717-727. 10.1002/1873-3468.13640 31627256 PMC7042088

[DEV201370C87] Pravata, V. M., Omelková, M., Stavridis, M. P., Desbiens, C. M., Stephen, H. M., Lefeber, D. J., Gecz, J., Gundogdu, M., Õunap, K., Joss, S., et al. (2020b). An intellectual disability syndrome with single-nucleotide variants in O-GlcNAc transferase. *Eur. J. Hum. Genet.* 28, 706-714. 10.1038/s41431-020-0589-9 32080367 PMC7253464

[DEV201370C88] Radermacher, P. T., Myachina, F., Bosshardt, F., Pandey, R., Mariappa, D., Müller, H.-A. J. and Lehner, C. F. (2014). O-GlcNAc reports ambient temperature and confers heat resistance on ectotherm development. *Proc. Natl. Acad. Sci USA* 111, 5592-5597. 10.1073/pnas.1322396111 24706800 PMC3992692

[DEV201370C89] Ruan, H. B., Nie, Y. and Yang, X. (2013). Regulation of protein degradation by O-GlcNAcylation: crosstalk with ubiquitination. *Mol. Cell. Proteomics* 12, 3489-3497. 10.1074/mcp.R113.029751 23824911 PMC3861702

[DEV201370C90] Saklayen, M. G. (2018). The global epidemic of the metabolic syndrome. *Curr. Hypertens. Rep.* 20, 12. 10.1007/s11906-018-0812-z 29480368 PMC5866840

[DEV201370C91] Schnerch, A., Cerdan, C. and Bhatia, M. (2010). Distinguishing between mouse and human pluripotent stem cell regulation: the best laid plans of mice and men. *Stem Cells* 28, 419-430. 10.1002/stem.298 20054863

[DEV201370C92] Selvan, N., Williamson, R., Mariappa, D., Campbell, D. G., Gourlay, R., Ferenbach, A. T., Aristotelous, T., Hopkins-Navratilova, I., Trost, M. and Van Aalten, D. M. F. (2017). A mutant O-GlcNAcase enriches Drosophila developmental regulators. *Nat. Chem. Biol.* 13, 882-887. 10.1038/nchembio.2404 28604694 PMC7611224

[DEV201370C93] Shafi, R., Iyer, S. P. N., Ellies, L. G., O'Donnell, N., Marek, K. W., Chui, D., Hart, G. W. and Marth, J. D. (2000). The O-GlcNAc transferase gene resides on the X chromosome and is essential for embryonic stem cell viability and mouse ontogeny. *Proc. Natl. Acad. Sci. USA* 97, 5735-5739. 10.1073/pnas.100471497 10801981 PMC18502

[DEV201370C94] Shao, M. S., Yang, X., Zhang, C. C., Jiang, C. Y., Mao, Y., Xu, W. D., Ma, L. and Wang, F. F. (2022). O-GlcNAcylation in ventral tegmental area dopaminergic neurons regulates motor learning and the response to natural reward. *Neurosci. Bull.* 38, 263-274. 10.1007/s12264-021-00776-8 34741260 PMC8975958

[DEV201370C95] Shen, H., Zhao, X., Chen, J., Qu, W., Huang, X., Wang, M., Shao, Z., Shu, Q. and Li, X. (2021). O-GlcNAc transferase Ogt regulates embryonic neuronal development through modulating Wnt/β-catenin signaling. *Hum. Mol. Genet.* 31, 57-68. 10.1093/hmg/ddab223 34346496

[DEV201370C96] Shi, F. T., Kim, H., Lu, W., He, Q., Liu, D., Goodell, M. A., Wan, M. and Songyang, Z. (2013). Ten-eleven translocation 1 (Tet1) is regulated by o-linked n-acetylglucosamine transferase (ogt) for target gene repression in mouse embryonic stem cells. *J. Biol. Chem.* 288, 20776-20784. 10.1074/jbc.M113.460386 23729667 PMC3774349

[DEV201370C97] Sinclair, D. A. R., Syrzycka, M., Macauley, M. S., Rastgardani, T., Komljenovic, I., Vocadlo, D. J., Brock, H. W. and Honda, B. M. (2009). Drosophila O-GlcNAc transferase (OGT) is encoded by the Polycomb group (PcG) gene, super sex combs (sxc). *Proc. Natl. Acad. Sci. USA* 106, 13427-13432. 10.1073/pnas.0904638106 19666537 PMC2726349

[DEV201370C98] Sohn, K. C., Lee, E. J., Shin, J. M., Lim, E. H., No, Y., Lee, J. Y., Yoon, T. Y., Lee, Y. H., Im, M., Lee, Y., et al. (2014). Regulation of keratinocyte differentiation by O-GlcNAcylation. *J. Dermatol. Sci.* 75, 10-15. 10.1016/j.jdermsci.2014.04.010 24802710

[DEV201370C99] Speakman, C. M., Domke, T. C. E., Wongpaiboonwattana, W., Sanders, K., Mudaliar, M., Van Aalten, D. M. F., Barton, G. J. and Stavridis, M. P. (2014). Elevated *O*-GlcNAc levels activate epigenetically repressed genes and delay mouse ESC differentiation without affecting naïve to primed cell transition. *Stem Cells* 32, 2605-2615. 10.1002/stem.1761 24898611 PMC4737245

[DEV201370C204] Struhl, G. and Akam, M. (1985). Altered distributions of Ultrabithorax transcripts in extra sex combs mutant embryos of Drosophila. *EMBO J.* 4, 3259-3264. 10.1002/j.1460-2075.1985.tb04075.x 2419125 PMC554652

[DEV201370C100] Swamy, M., Pathak, S., Grzes, K. M., Damerow, S., Sinclair, L. V., Van Aalten, D. M. F. and Cantrell, D. A. (2016). Glucose and glutamine fuel protein O-GlcNAcylation to control T cell self-renewal and malignancy. *Nat. Immunol.* 17, 712-720. 10.1038/ni.3439 27111141 PMC4900450

[DEV201370C101] Tahiliani, M., Koh, K. P., Shen, Y., Pastor, W. A., Bandukwala, H., Brudno, Y., Agarwal, S., Iyer, L. M., Liu, D. R., Aravind, L., et al. (2009). Conversion of 5-methylcytosine to 5-hydroxymethylcytosine in mammalian DNA by MLL partner TET1. *Science* 324, 930-935. 10.1126/science.1170116 19372391 PMC2715015

[DEV201370C102] Tan, Z. W., Fei, G., Paulo, J. A., Bellaousov, S., Martin, S. E. S., Duveau, D. Y., Thomas, C. J., Gygi, S. P., Boutz, P. L. and Walker, S. (2021). O-GlcNAc regulates gene expression by controlling detained intron splicing. *Nucleic Acids Res.* 48, 5656-5669. 10.1093/nar/gkaa263 PMC7261177 32329777

[DEV201370C103] Torres, C. R. and Hart, G. W. (1984). Topography and polypeptide distribution of terminal N-acetylglucosamine residues on the surfaces of intact lymphocytes. Evidence for O-linked GlcNAc. *J. Biol. Chem.* 259, 3308-3317. 10.1016/S0021-9258(17)43295-9 6421821

[DEV201370C104] Vaidyanathan, K., Niranjan, T., Selvan, N., Teo, C. F., May, M., Patel, S., Weatherly, B., Skinner, C., Opitz, J., Carey, J., et al. (2017). Identification and characterization of a missense mutation in the O-linked β-N-acetylglucosamine (O-GlcNAc) transferase gene that segregates with X-linked intellectual disability. *J. Biol. Chem.* 292, 8948-8963. 10.1074/jbc.M116.771030 28302723 PMC5448127

[DEV201370C105] Varki, A. (2017). Biological roles of glycans. *Glycobiology* 27, 3-49. 10.1093/glycob/cww086 27558841 PMC5884436

[DEV201370C106] Wan, L., Xu, K., Wei, Y., Zhang, J., Han, T., Fry, C., Zhang, Z., Wang, Y. V., Huang, L., Yuan, M., et al. (2018). Phosphorylation of EZH2 by AMPK suppresses PRC2 methyltransferase activity and oncogenic function. *Mol. Cell* 69, 279-291.e5. 10.1016/j.molcel.2017.12.024 29351847 PMC5777296

[DEV201370C107] Watson, L. J., Long, B. W., Demartino, A. M., Brittian, K. R., Readnower, R. D., Brainard, R. E., Cummins, T. D., Annamalai, L., Hill, B. G. and Jones, S. P. (2014). Cardiomyocyte Ogt is essential for postnatal viability. *Am. J. Physiol. Heart Circ. Physiol.* 306, H142. 10.1152/ajpheart.00438.2013 24186210 PMC3920156

[DEV201370C108] Webster, D. M., Teo, C., Sun, Y., Wloga, D., Gay, S., Klonowski, K. D., Wells, L. and Dougan, S. T. (2009). O-GlcNAc modifications regulate cell survival and epiboly during zebrafish development. *BMC Dev. Biol.* 9, 28. 10.1186/1471-213X-9-28 19383152 PMC2680843

[DEV201370C109] Whelan, S. A., Lane, M. D. and Hart, G. W. (2008). Regulation of the O-linked beta-N-acetylglucosamine transferase by insulin signaling. *J. Biol. Chem.* 283, 21411-21417. 10.1074/jbc.M800677200 18519567 PMC2490780

[DEV201370C110] White, C. W., Fan, X., Maynard, J. C., Wheatley, E. G., Bieri, G., Couthouis, J., Burlingame, A. L. and Villeda, S. A. (2020). Age-related loss of neural stem cell O-GlcNAc promotes a glial fate switch through STAT3 activation. *Proc. Natl. Acad. Sci. USA* 117, 22214-22224. 10.1073/pnas.2007439117 32848054 PMC7486730

[DEV201370C111] Willems, A. P., Gundogdu, M., Kempers, M. J. E., Giltay, J. C., Pfundt, R., Elferink, M., Loza, B. F., Fuijkschot, J., Ferenbach, A. T., Van Gassen, K. L. I., et al. (2017). Mutations in *N*-acetylglucosamine (*O*-GlcNAc) transferase in patients with X-linked intellectual disability. *J. Biol. Chem.* 292, 12621-12631. 10.1074/jbc.M117.790097 28584052 PMC5535036

[DEV201370C112] Wong, K. K. L., Liu, T. W., Parker, J. M., Sinclair, D. A. R., Chen, Y. Y., Khoo, K. H., Vocadlo, D. J. and Verheyen, E. M. (2020). The nutrient sensor OGT regulates Hipk stability and tumorigenic-like activities in Drosophila. *Proc. Natl. Acad. Sci. USA* 117, 2004-2013. 10.1073/pnas.1912894117 31932432 PMC6994980

[DEV201370C113] Wulff-Fuentes, E., Berendt, R. R., Massman, L., Danner, L., Malard, F., Vora, J., Kahsay, R. and Olivier-Van Stichelen, S. (2021). The human O-GlcNAcome database and meta-analysis. *Sci. Data* 8, 25. 10.1038/s41597-021-00810-4 33479245 PMC7820439

[DEV201370C114] Xiong, X., Ma, H., Ma, J., Wang, X., Li, D. and Xu, L. (2022). αSMA-Cre-mediated Ogt deletion leads to heart failure and vascular smooth muscle cell dysfunction in mice. *Biochem. Biophys. Res. Commun.* 625, 31-37. 10.1016/j.bbrc.2022.07.106 35944361

[DEV201370C115] Xu, Q., Yang, C., Du, Y., Chen, Y., Liu, H., Deng, M., Zhang, H., Zhang, L., Liu, T., Liu, Q., et al. (2014). AMPK regulates histone H2B O-GlcNAcylation. *Nucleic Acids Res.* 42, 5594-5604. 10.1093/nar/gku236 24692660 PMC4027166

[DEV201370C116] Yang, X., Su, K., Roos, M. D., Chang, Q., Paterson, A. J. and Kudlow, J. E. (2001). O-linkage of N-acetylglucosamine to Sp1 activation domain inhibits its transcriptional capability. *Proc. Natl. Acad. Sci. USA* 98, 6611-6616. 10.1073/pnas.111099998 11371615 PMC34401

[DEV201370C117] Yang, Y. R., Song, M., Lee, H., Jeon, Y., Choi, E.-J. J., Jang, H.-J. J., Moon, H. Y., Byun, H.-Y. Y., Kim, E.-K. K., Kim, D. H., et al. (2012) O-GlcNAcase is essential for embryonic development and maintenance of genomic stability. *Aging Cell* 11, 439-448. 10.1111/j.1474-9726.2012.00801.x 22314054

[DEV201370C118] Yang, Y. R., Jang, H. J., Lee, Y. H., Kim, I. S., Lee, H., Ryu, S. H. and Suh, P. G. (2015). O-GlcNAc cycling enzymes control vascular development of the placenta by modulating the levels of HIF-1α. *Placenta* 36, 1063-1068. 10.1016/j.placenta.2015.08.001 26286378

[DEV201370C119] Zachara, N. E., O'Donnell, N., Cheung, W. D., Mercer, J. J., Marth, J. D. and Hart, G. W. (2004). Dynamic O-GlcNAc modification of nucleocytoplasmic proteins in response to stress: a survival response of mammalian cells. *J. Biol. Chem.* 279, 30133-30142. 10.1074/jbc.M403773200 15138254

[DEV201370C120] Zhang, Z., Parker, M. P., Graw, S., Novikova, L. V., Fedosyuk, H., Fontes, J. D., Koestler, D. C., Peterson, K. R. and Slawson, C. (2019). O-GlcNAc homeostasis contributes to cell fate decisions during hematopoiesis. *J. Biol. Chem.* 294, 1363-1379. 10.1074/jbc.RA118.005993 30523150 PMC6349094

[DEV201370C121] Zhu, Q., Cheng, X., Cheng, Y., Chen, J., Xu, H., Gao, Y., Duan, X., Ji, J., Li, X. and Yi, W. (2020). O-GlcNAcylation regulates the methionine cycle to promote pluripotency of stem cells. *Proc. Natl. Acad. Sci. USA* 117, 7755-7763. 10.1073/pnas.1915582117 32193337 PMC7148567

